# Youth Epidemiology and Resilience (YEAR) in a student population: prevalence and predictors of mental health symptoms

**DOI:** 10.3389/fpsyt.2024.1454484

**Published:** 2024-11-01

**Authors:** John Chee Meng Wong, Natalie Cheok Ling Lei, Dennis Mun Yen Kom, Victoria Hui Fen Fee, Natalie Huijing Yap, Jie Yu Teoh, Liang Shen, Qai Ven Yap, Michelle Jing Si Wan, Ruochen Du, Leoniek Kroneman, Daniel Shuen Sheng Fung, Say How Ong, Cheong Sing Tian, Muhammad Nabil Syukri Bin Sachiman, Nicholas En-Ping Sii, Jia Ying Teng, Tze Pin Ng, Frank Verhulst

**Affiliations:** ^1^ Department of Psychological Medicine, Yong Loo Lin School of Medicine, National University of Singapore, Singapore, Singapore; ^2^ Department of Psychological Medicine, National University Health System, Singapore, Singapore; ^3^ Student Development Curriculum Division, Ministry of Education, Singapore, Singapore; ^4^ National University Singapore Medicine Biostatistics Unit, National University of Singapore, Singapore, Singapore; ^5^ Department of Developmental Psychiatry, Institute of Mental Health, Singapore, Singapore; ^6^ Department of Child and Adolescent Psychiatry, Erasmus University Medical Center, Rotterdam, Netherlands

**Keywords:** mental health, resilience, adolescence, epidemiology, demographics

## Abstract

**Background:**

Adolescence is a pivotal developmental phase, marked by a high prevalence of mental health issues. The Singapore Youth Epidemiology and Resilience (YEAR) study aims to assess the prevalence of mental health symptoms, sociodemographic risk factors, and the protective role of resilience in a multi-ethnic, Asian school-going adolescent population.

**Methods:**

School-based adolescents aged 11-18 and their parents were invited from a stratified sample of national schools, designed to be demographically representative of the mainstream school-going population. In the screening phase, adolescents completed a battery of questionnaires including the Youth Self-Report (YSR), Child Behaviour Checklist (CBCL), and the Singapore Youth Resilience Scale (SYRESS). Thereafter, diagnostic interviews were conducted on high-risk and control participants. Data analysis was conducted to establish prevalence rates of mental health symptoms, and to examine the associations between mental health symptoms, sociodemographic factors, and resilience.

**Results:**

The final study sample consisted of 3336 adolescents and 2475 parents. Based on adolescents’ response on the YSR, 37.3%, 13.1% and 27.7% of the YEAR Study population scored in the clinical range for internalizing, externalizing and total problems respectively. Based on parents’ reporting on their children on the CBCL, 15.9%, 4.0% and 11.2% scored in the clinical range for internalizing, externalizing and total problems respectively. Sociodemographic risk factors for specific mental health symptoms were identified, including belonging to the age category of 15-16 (OR, 1.8-2.2) and those living in either a 4-, 5-room or executive flat (OR, 0.6-0.7), or in condominiums and other apartments (OR, 0.4-0.6). Total resilience scores were moderately correlated with total [rs(3334) = -.49, p <.01] and internalizing [rs(3334) = -.50, p <.01] problem scores on the YSR.

**Conclusion:**

This study illustrates the state of mental health of school-going adolescents aged 11-18. The greater prevalence of internalizing symptoms indicates the need for stronger attention for identifying internalizing problems and targeted interventions for those at risk of such symptoms. The association between mental health symptoms and resilience factors highlights the protective potential of resilience building for adolescents.

## 1 Introduction

Adolescence is a crucial developmental phase, broadly understood as the transitory period between childhood and adulthood, during which individuals undergo their formative years of physical, socio-emotional and identity development (1).

Due to the significant changes in brain systems related to higher cognitive functions, emotional control, and risk-reward assessment during adolescence, such developmental challenges may increase the risk of developing mental health disorders if insufficiently addressed (2). According to the World Health Organisation (WHO), one in seven 10- to 19-year-olds suffer from a mental health condition globally. Furthermore, mental health conditions contribute to 16% of the global disease burden for this age group (3). Yet, mental health conditions in adolescents are often undetected and untreated, due to stigmatisation (4) and low mental health literacy (5), the latter defined as “understanding how to obtain and maintain positive mental health; understanding mental disorders and their treatments; decreasing stigma related to mental disorders; and, enhancing help-seeking efficacy” (6).

Based on a local recent web survey conducted in Singapore from April to June 2022, 11.7% and 12.8% of children and youth had depressive and anxiety symptoms respectively based on parental responses (7), indicating an urgent need to understand and address the mental health status and resilience of our adolescents. National figures on the prevalence and incidence of psychiatric disorders in adolescents are useful for the implementation of change-affecting policies, establishing research priorities, and monitoring the effect of changes in treatment or service funding. Although the Singapore Mental Health Study (SMHS) provides figures for Singaporeans aged 18 and older (8, 9), no such data is currently available for school-going Singaporean adolescents. Furthermore, although Chodavadia et al. (7)’s study provides recent national data, it leverages on proxy reports from parents, who may be more likely to report more severe cases. The current study employs self-report in addition to parent-report, building upon Chodavadia et al. (7)’s findings by increasing sensitivity of identifying cases, especially with respect to internalizing symptoms such as anxiety and depression. As noted in literature, parents are more likely to report externalizing symptoms in their child as opposed to internalizing symptoms (10).

In addition to establishing prevalence, examining resilience in relation to mental health disorders would help to provide a more holistic picture of mental health (11) and better inform both preventative measures and psychological interventions for adolescents. Resilience is defined as a dynamic process or capacity to adapt and “bounce back” from adversities or life challenges (12–14), a multi-dimensional trait that changes with different demographic factors (e.g., age, gender, socioeconomic status, and ethnicity) and the circumstances that individuals are in and subjected to (15). Being malleable, resilience can be built upon by improving resilience-based skills such as mindfulness and/or cognitive-behavioural skills (16). In recent years, resilience studies have been gaining traction in understanding the risks of developing psychopathologies – many have found resilience to be negatively associated with depression and anxiety (17–19), even exerting protective or buffering effects (20, 21).

Considering the above, the Singapore Youth Epidemiology and Resilience (YEAR) study was conceived to establish the prevalence rates of psychiatric disorders and reflect the state of resilience for school-going adolescents aged 11-18. Additionally, it aims to assess the levels of psychosocial, familial, and academic stress and their relationships to adolescent mental health, as well as examine how resilience modulates the effects of stress on mental health. Investigating adolescent identity development and impairment and its age trajectory is also a key objective. Ultimately, the study strives to accurately provide the above information for policy makers to aid in their decision-making to raise public awareness of adolescent mental health issues, and to plan, design and implement school-based interventions to detect, treat and prevent such problems.

This present article will focus on the: (i) study methodology, (ii) reporting of prevalence rates of mental health symptoms, (iii) identification of sociodemographic risk factors for five DSM-oriented mental health symptom clusters (affective problems, anxiety problems, somatic problems, attention-deficit hyperactive problems, oppositional-defiant/conduct problems) and comorbidities, as well as the (iv) association between resilience factors and mental health symptoms. The prevalence rates of mental health disorders and associated risk factors will be discussed in a subsequent manuscript.

To summarise, the research questions of this current study include:

1. What are current prevalence rates of mental health symptoms in 11- to 18-year-olds in Singapore? What are the rates of comorbidity?
2. What are some sociodemographic risk factors of mental health symptoms?
3. How is adolescents’ resilience related to the presence (or absence) of mental health symptoms?

## 2 Methods

### 2.1 Sample

The YEAR Study is a cross-sectional epidemiological study (September 2018 – present). A school-based study design was chosen as most typically developing children in Singapore are in the public education system. According to data from The United Nations Educational, Scientific and Cultural Organization (UNESCO), 99.4% of all Singaporean children were enrolled in primary education in 2021 (22). The school-based approach also maximises the response rate by leveraging the organisational structure. The sampling frame consisted of 40 schools stratified by level; primary, secondary and post-secondary, zone, and type, excluding special education schools. 16,000 students aged 11-18 and their parents were randomly selected and approached to participate in the study. The sample was designed to be nationally representative of the mainstream school-going population in terms of sex, ethnicity, father’s education level, mother’s education level and housing type. Physical or Electronic Participant Information Sheets and Consent forms were distributed to the invited students, and consent from parent and assent from child was taken. 20.9% (n = 3336) of the invited students consented to the study. The consent rate is in line with other national school studies conducted during the COVID-19 pandemic.

### 2.2 Procedure

Survey information was gathered after consent taking in two-stages: initial questionnaire assessment, and subsequent in-person diagnostic interview of screened high-risk participants and controls. In Stage 1, consented students completed the online questionnaires either in school or at home. To assure the quality of data collected, members of the research team were present during the school screenings and closely supervised the survey respondents. For respondents with clarifications on the questionnaire, study team members were present and assisted them accordingly during in-person school screenings. For online screenings, respondents were able to clarify any queries *via* a chatbot manned by a study team member. The questionnaire was also pilot-tested in a smaller sample of school-going adolescents of the same age range to ensure readability. 2475 consented parents and 958 consented teachers of the respective students completed a battery of questionnaires (see Appendix). To identify subjects for Stage 2, the procedure described by Verhulst & Van der Ende (23) was adapted. The average 75th percentile of the cumulative frequency distribution of the mean screening z-scores of the child’s Youth Self-Report (YSR) Total Problem score was used as a cutoff score to select participants. Individuals who scored in the top 25th percentile, as well as the first 100 of the 10% of the remaining with normal scores were selected and invited to receive the semi-structured Kiddie Schedule for Affective Disorders and Schizophrenia Present and Lifetime Version (K-SADS-PL) screen interview and complete a battery of questionnaires (see Appendix). The stage 2 sample comprised of 474 adolescents. Consented parents and adolescents were interviewed either face-to-face or online separately. Interviewers were trained by an external trainer from the University of Pittsburgh, where the K-SADS was developed. Interviews were audio-recorded and randomly reviewed by a senior research team member for quality assurance. This manuscript only reports findings from stage 1.

#### 2.2.1 Safety risk protocol

All the participants who reported either having had thoughts or intention to harm or kill themselves on the YSR were contacted and invited for the Stage 2 interview as a safety protocol. For those who were found to be at-risk during the interview, a list of counselling hotlines and resources was provided for them to seek help. This included several outpatient and community services. Interviewers also suggested approaching school counsellor/counselling services. If the parent and child had further enquiries or consented to a referral, the team would recommend other private practitioners or facilitate the referral to school counsellors or REACH services – a community-based mental healthcare service for students. For at-risk participants who declined to participate in the interview or were uncontactable, a brochure with self-care resources was provided *via* email.

### 2.3 Measures

#### 2.3.1 Youth self-report and child behavior checklist

The Youth Self-Report (YSR) and Child Behavior Checklist for Ages 6 to 18 (CBCL) are established measures of emotional and behavioural problems among adolescents. The items within the two questionnaires mirror each other; while the YSR is completed by the adolescent, the CBCL is rated by their parent. The YSR and CBCL consist of 112 and 113 items respectively, scored based on experiences in the past six months according to a 3-point Likert scale: 0 (Not True), 1 (Somewhat or Sometimes True), and 2 (Very and Often True). The ratings are summarised in three broad composite scores (total problems score, internalizing problem score, and externalizing problem score), with eight empirically-derived subscale scores (i.e., anxious/depressed, depressed/withdrawn, somatic complaints, attention problems, social problems, thought problems, aggressive behavior, and rule-breaking behavior). Separately, the data can also be presented and analysed using six DSM-oriented scales (i.e., affective, anxiety, somatic, attention-deficit hyperactive, oppositional-defiant, and conduct problems). The good validity and reliability of the YSR and CBCL are well documented by Achenbach & Rescorla (24). In this study, Cronbach’s αs for the internalizing, externalizing and total problems scales were 0.78, 0.87 and 0.73 respectively for the YSR. For the empirically-derived scales, Cronbach’s αs ranged from 0.71 to 0.87. For the DSM-oriented scales, Cronbach’s αs ranged from 0.70 to 0.83. For the CBCL, good internal consistencies for the composite scales were reported (α = 0.89; 0.89; 0.93 for internalizing, externalizing and total problems respectively). For the empirically-derived scales, Cronbach’s αs ranged from 0.69 to 0.86. For the DSM-oriented scales, Cronbach’s α ranged from 0.63 to 0.80.

The YSR and CBCL can also be categorised into three severity ranges – normal, borderline and clinical – using cut-offs based on international norms. Scores in the normal range are considered within typical range of adolescents’ functioning. Scores in the borderline category suggest a higher risk of mental health problems but are not within clinical range. Scores in the clinical range indicate mental health problems comparable to those referred for professional help within mental health services. The terminology used in this manuscript has been aligned with the instrument manual (24) for the YSR and CBCL, for purpose of consistency.

#### 2.3.2 Singapore youth resilience scale

The Singapore Youth Resilience Scale (SYRESS) is a self-reported, 50-item scale that measures the multidimensional construct of resilience among Singaporean adolescents, constructed and validated based on the contextual and cultural aspects of the Singaporean adolescent population. Comprising of 10 domains – Perseverance/commitment, Positive Self-Image/Optimism, Relationship/Social Support, Humour/Positive Thinking, Emotional Regulation, Spirituality/Faith, Personal Confidence/Responsibility, Personal Control, Flexibility, and Positive Coping, the good validity and reliability of the SYRESS was documented by Lim et al. (25). In this study, the Cronbach α for the overall scale was 0.95. The Cronbach’s α for most of the subscales ranged from 0.43-0.85. The items are rated on a 5-point Likert scale: 1 (Never), 2 (Sometimes), 3 (About half the time), 4 (Most of the time) and 5 (Always).

English versions of the above scales were used in this study.

#### 2.3.3 Sociodemographic factors

Sociodemographic factors were sex (male or female), age, ethnicity, type of housing and parent’s highest level of education, parent’s gender, presence of domestic helper (present/absent), household composition, number of siblings, and child’s medical conditions.

Age was categorised as the following: 11-12, 13-14, 15-16, 17-18. Ethnicity was categorised as: Chinese, Malay, Indian or Other (comprises ethnicities not listed in the first three categories). For type of housing, the categories are Housing and Development Board (HDB) 1-, 2-, or 3-room flat, 4-, 5-room or executive flat, condominiums or other apartments, and landed property. HDB flats are public housing flats built by the Singapore government for Singaporeans and Permanent Residents. Executive flats contain an easily convertible additional space (e.g., balcony) compared to other HDB flats. The categories of the parent’s highest education level are adapted from the Singapore Standard Educational Classification (SSEC) – Primary, Secondary, Post-secondary, Diploma and Professional Attainment, and University. Post-secondary refers to non-tertiary general and vocational education level, such as junior colleges and national Institute of Technical Education certificates (NITECs). Diploma and professional attainment may include polytechnic diplomas and qualifications awarded by professional bodies. University level refers to Bachelor’s, Master’s or Doctorate degrees or postgraduate diploma or certificates. Household composition was categorised as 2 parents + child(ren), 1 parent + child(ren), parent(s) + child(ren) + grandparent(s), and Grandparent(s) + Child(ren) or Others. Child medical conditions surveyed were asthma, diabetes, being overweight and underweight.

### 2.4 Data management and statistical analysis

All consented participants were assigned a non-identifiable ID. Subsequently, collected data were anonymised using their non-identifiable ID to protect participant privacy and confidentiality. To minimise transcription errors in data collection and processing, an online survey platform (Qualtrics) was used to record all responses.

Data were analysed using IBM Statistical Package for the Social Sciences (SPSS, version 29.0.1.0). Descriptive analyses were conducted to derive percentages of individuals who fell in the normal, borderline, and clinical ranges on the YSR and CBCL, as well as the sociodemographic breakdown for individuals who met clinical range cut-offs for the DSM-oriented scales of the YSR and CBCL. Borderline and clinical range cut-offs used were Group 2 norms derived and advised by Achenbach & Rescorla (26) for countries who have yet to derive their own norms. To better reflect the general population distribution, prevalence estimates for the CBCL were weighted to adjust for sex, type of housing and parent’s highest education level (27). Prevalence estimates for the YSR were weighted to adjust for sex. Binary logistic regression analyses were conducted to examine the association between sociodemographic variables and the DSM-oriented YSR and CBCL scales, as well as the association between sociodemographic variables and the presence of comorbidity. Only certain sociodemographic variables were included in the logistic regression analyses due to multicollinearity, namely age, sex, ethnicity, type of housing and parent’s highest education level. For the DSM-oriented scales, oppositional-defiant and conduct problems were grouped into one variable in regression analyses, as they lie on the same diagnostic spectrum. Comorbidity was operationalised as meeting the clinical cut-off for more than one of the five DSM-oriented scales. Non-parametric correlation analyses were conducted to examine the association between adolescent problem scores on the broad-band YSR scales and resilience scores.

## 3 Results

### 3.1 Sample characteristics

**Table 1** depicts the sociodemographic distribution of the sample. In comparison with national data, the demographic profile of the YEAR Study is largely representative of the adolescent and the population demographic distribution in the general population in terms of age and ethnicity, in comparison with data from the Department of Statistics of Singapore. Sex, housing type and parents’ highest level of education were not proportionally representative of the local adolescents’ population profile based on comparison with Department of Statistics data.

**Table 1:** Sociodemographic distribution of the sample.

Sociodemographics		n	% of YEAR Study Population	% of National Population
**Age Group (years)**	11-12	977	29.3	25.6
**(Mean = 14)**	13-14	911	27.3	25.1
	15-16	714	21.4	24.0
	17-18	734	22.0	25.1
Sex*	Female	1816	54.4	49.0
	Male	1520	45.6	51.0
**Ethnicity**	Chinese	2337	70.1	74.2
	Indian	371	11.1	8.9
	Malay	483	14.5	13.7
	Others	145	4.3	3.2
Type of Housing*	HDB 1-, 2, or 3-room flat	318	12.8	24.1
	HDB 4- or 5-room or executive flat	1486	60.0	54.2
	Condominiums or other apartments	536	21.7	16.5
	Landed property	135	5.5	4.9
Parent’s Highest Education Level*	Below secondary	92	3.7	21.6
	Secondary	408	16.5	16.5
	Post-secondary	184	7.4	9.4
	Diploma & professional attainment	654	26.4	16.3
	University	1137	45.9	36.1
**Parent’s Gender**	Female	1802	72.8	–
	Male	673	27.2	–
**Presence of Domestic Helper**	Yes	605	24.4	–
	No	1870	75.6	–
**Household Composition**	2 parents + child(ren)	1777	71.8	–
	1 parent + child(ren)	188	7.6	–
	parent(s) + child(ren) + grandparent(s)	453	18.3	–
	Grandparent(s) + Child(ren) or Others	57	2.3	–
**Number of Siblings** **(Mean = 1.44, SD = 1.04)**	–	–	–	–
**Child's Medical Conditions**	Asthma (Yes)	98	4.0	–
	Asthma (No)	2377	96.0	–
	Diabetes (Yes)	3	0.1	–
	Diabetes (No)	2472	99.1	–
	Overweight (Yes)	314	12.7	–
	Overweight (No)	2161	87.3	–
	Underweight (Yes)	184	7.4	–
	Underweight (No)	2291	92.6	–

*Variables whose proportions exceed a 5-point deviation from local adolescents’ population profile based on comparison with Department of Statistics of Singapore data.

### 3.2 Prevalence of mental health symptoms (self- and parent report)

**Table 2** shows the unweighted and weighted prevalence of mental health symptoms in the population based on adolescent self-report using the YSR, and parent report using the CBCL. On the YSR, internalizing problems were most prevalent, with 37.3% adolescents meeting the clinical cut-off, compared to externalizing and total problems (13.1% and 27.7% respectively). Under the empirically-derived YSR subscales, 20.3% of adolescents had scores in the clinical range on the anxious/depressed scale, 14.5% on the withdrawn/depressed scale, and 15.1% with social problems scores in the clinical range. For the DSM-oriented scales, 41.5% of adolescents had anxiety problems scores in the clinical range, followed by 15% who had affective problems in the clinical range. On the CBCL, internalizing problems were most prevalent, with 15.9% adolescents meeting the clinical range cut-off, compared to externalizing and total problems (4.0% and 11.2% respectively). 6.5% of adolescents had scores in the clinical range on the somatic problems scale, followed by 5.0% who had anxious/depressed scores in the clinical range. For the DSM-oriented scales, 15.9% of adolescents had anxiety problems scores in the clinical range, followed by 4.8% who had affective problems scores in the clinical range.

**Table 2:** Prevalence of Adolescents’ Problem Scores based on the YSR and CBCL.

YSR									
	Normal			Borderline			Clinical		
	*n*	unweighted (%)	weighted (%)	*n*	unweighted (%)	weighted (%)	*n*	unweighted (%)	weighted (%)
Broad-band scales									
Internalizing	1573	47.2	47.1	521	15.6	15.6	1242	37.2	37.3
Externalizing	2580	77.3	77.3	326	9.8	9.6	430	12.9	13.1
Total Problems	1879	56.3	56.2	538	16.1	16.1	919	27.5	27.7
Empirically-based scales									
Anxious/Depressed	2146	64.3	64.4	510	15.3	15.4	680	20.4	20.3
Withdrawn/Depressed	2328	69.8	69.7	524	15.7	15.7	484	14.5	14.5
Somatic Complaints	2702	81.0	81.3	362	10.9	10.2	272	8.2	8.5
Social Problems	2381	71.4	71.5	446	13.4	13.4	509	15.3	15.1
Thought Problems	2761	82.8	82.8	258	7.7	7.5	317	9.5	9.7
Attention Problems	2588	77.6	77.7	398	11.9	12.0	350	10.5	10.4
Rule breaking behavior	3150	94.4	94.4	143	4.3	4.2	43	1.3	1.4
Aggressive behavior	2821	84.6	84.3	295	8.8	8.9	220	6.6	6.9
DSM-Oriented scales									
Affective Problems	2245	67.3	67.4	589	17.7	17.5	502	15.0	15.1
Anxiety Problems	1592	47.7	48.0	352	10.6	10.5	1392	41.7	41.5
Somatic Problems	2788	83.6	83.2	348	10.4	10.6	200	6.0	6.2
ADHD Problems	2785	83.5	83.5	353	10.6	10.6	198	5.9	5.9
Oppositional Defiant Problems	2900	86.9	87.0	218	6.5	6.5	218	6.5	6.5
Conduct Problems	3001	90.0	89.9	198	5.9	5.9	137	4.1	4.2
CBCL									
Broad-band scales									
Internalizing	1900	76.8	75.5	218	8.8	8.5	357	14.4	15.9
Externalizing	2315	93.5	92.5	87	3.5	3.4	73	2.9	4.0
Total Problems	2050	82.8	80.5	182	7.4	8.3	243	9.8	11.2
Empirically-based scales									
Anxious/Depressed	2205	89.1	87.9	174	7.0	7.6	96	3.9	4.5
Withdrawn/Depressed	2155	87.1	87.7	190	7.7	7.3	130	5.3	5.0
Somatic Complaints	2234	90.3	88.8	117	4.7	4.7	124	5.0	6.5
Social Problems	2219	89.7	88.0	180	7.3	9.0	76	3.1	2.9
Thought Problems	2215	89.5	88.7	160	6.5	6.7	100	4.0	4.6
Attention Problems	2242	90.6	90.4	159	6.4	6.4	74	3.0	3.1
Rule breaking behavior	2424	97.9	97.0	33	1.3	1.7	18	0.7	1.3
Aggressive behavior	2372	95.8	94.8	63	2.5	3.9	40	1.6	1.3
DSM-Oriented scales									
Affective Problems	2200	88.9	87.4	172	6.9	7.8	103	4.2	4.8
Anxiety Problems	1937	78.3	76.8	183	7.4	7.3	355	14.3	15.9
Somatic Problems	2303	93.1	92.0	80	3.2	3.5	92	3.7	4.5
ADHD Problems	2261	91.4	90.7	177	7.2	7.4	37	1.5	2.0
Oppositional Defiant Problems	2357	95.2	94.4	86	3.5	4.5	32	1.3	1.1
Conduct Problems	2390	96.6	96.1	63	2.6	2.9	22	0.9	1.0

Prevalence estimates on the YSR are weighted by sex, prevalence estimates on the CBCL are weighted by sex, type of housing and parent’s highest education level.

**Table 3** illustrates the distribution of the prevalence of problem scores in the clinical range (as determined by the YSR and CBCL respectively) by age, sex, ethnicity, type of housing and parent’s highest educational level attained.

**Table 3:** Demographic Breakdown of individuals with DSM-Oriented Scale scores in the clinical range.

YSR												
	Affective Problems		Anxiety Problems		Somatic Problems		ADHD Problems		Oppositional Defiant/Conduct Problems		Comorbidities	
	*n*	%	*n*	%	*n*	%	*n*	%	*n*	%	*n*	*%*
Age Group												
11-12	100	10.2	355	36.3	72	7.4	42	4.3	76	7.8	149	15.3
13-14	136	14.9	366	40.2	51	5.6	62	6.8	75	8.2	171	18.8
15-16	148	20.7	364	51.0	47	6.6	49	6.9	79	11.1	173	24.2
17-18	118	16.1	307	41.8	30	4.1	45	6.1	67	9.1	145	19.8
Gender												
Male	232	15.3	599	39.4	84	5.5	85	5.6	139	9.1	296	19.5
Female	270	14.9	793	43.7	116	6.4	113	6.2	158	8.7	342	18.8
Ethnicity												
Chinese	313	13.4	961	41.1	125	5.3	137	5.9	188	8.0	407	17.4
Indian	37	10.0	128	34.5	11	3.0	12	3.2	23	6.2	50	13.5
Malay	123	25.5	242	50.1	55	11.4	39	8.1	76	15.7	149	30.8
Others	29	20.0	61	42.1	9	6.2	10	6.9	10	6.9	32	22.1
Type of Housing												
HDB 1-, 2- or 3-room flat	57	17.9	121	38.1	23	7.2	19	6.0	37	11.6	66	20.8
HDB 4- or 5-room flat or executive flat	213	14.3	634	42.7	82	5.5	92	6.2	133	9.0	286	19.2
Condominiums or other apartments	72	13.4	224	41.8	33	6.2	27	5.0	41	7.6	92	17.2
Landed property	17	12.6	52	38.5	6	4.4	7	5.2	6	4.4	21	15.6
Parent’s highest education level												
Below secondary	17	18.5	40	43.5	6	6.5	4	4.3	10	10.9	22	23.9
Secondary	70	17.2	183	44.9	25	6.1	32	7.8	39	9.6	91	22.3
Post-secondary	36	19.6	87	47.3	10	5.4	12	6.5	18	9.8	43	23.4
Diploma & professional attainment	99	15.1	260	39.8	36	5.5	40	6.1	65	9.9	127	19.4
University	137	12.0	461	40.5	67	5.9	57	5.0	85	7.5	182	16.0
CBCL												
Age Group												
11-12	70	9.6	182	24.9	50	6.8	74	10.1	56	7.7	111	15.2
13-14	75	10.7	167	23.9	42	6.0	78	11.2	54	7.7	113	16.2
15-16	79	14.6	120	22.2	48	8.9	40	7.4	33	6.1	87	16.1
17-18	51	10.1	69	13.7	32	6.4	22	4.4	19	3.8	47	9.3
Gender												
Male	117	10.6	281	25.3	74	6.7	138	12.4	91	8.2	184	16.6
Female	158	11.6	257	18.8	98	7.2	76	5.6	71	5.2	174	12.7
Ethnicity												
Chinese	197	11.1	393	22.1	119	6.7	158	8.9	128	7.2	263	14.8
Indian	28	10.5	48	18.0	15	5.6	19	7.1	8	3.0	28	10.5
Malay	41	13.0	71	22.5	28	8.9	31	9.8	21	6.6	50	15.8
Others	9	7.9	26	22.8	10	8.8	6	5.3	5	4.4	17	14.9
Type of Housing												
HDB 1-, 2- or 3-room flat	51	16.0	72	22.6	38	11.9	37	11.6	32	10.1	66	20.8
HDB 4- or 5-room flat or executive flat	164	11.0	330	22.2	96	6.5	115	7.7	101	6.8	208	14.0
Condominiums or other apartments	48	9.0	107	20.0	33	6.2	50	9.3	23	4.3	68	12.7
Landed property	12	8.9	29	21.5	5	3.7	12	8.9	6	4.4	16	11.9
Parent’s Highest Education Level												
Below secondary	14	15.2	22	23.9	8	8.7	9	9.8	9	9.8	13	14.1
Secondary	53	13.0	98	24.0	48	11.8	39	9.6	30	7.4	73	17.9
Post-secondary	24	13.0	49	26.6	15	8.2	18	9.8	12	6.5	32	17.4
Diploma & professional attainment	80	12.2	146	22.3	44	6.7	68	10.4	53	8.1	104	15.9
University	104	9.1	223	19.6	57	5.0	80	7.0	58	5.1	136	12.0

### 3.3 Sociodemographic correlates of clinical mental health symptoms

**Tables 4** and **5** present the results of the logistic regression analyses examining the association between sociodemographic predictors and the prevalence of adolescents’ problems. These analyses were adjusted for demographics.

**Table 4:** Adjusted sociodemographic correlates for adolescents’ problems in the clinical range (YSR).

	Affective Problems				Anxiety Problems				Somatic Problems			
	OR	95%		p value	OR	95%		p value	OR	95%		p value
		CI				CI				CI		
Age Group												
11-12 (reference)												
13-14	1.6	1.1	2.1	.007*	1.2	1	1.5	0.109	0.7	0.5	1.1	0.182
15-16	2.2	1.6	3	<.001**	1.9	1.5	2.4	<.001**	0.8	0.5	1.3	0.353
17-18	1.6	1.1	2.3	.007*	1.3	1	1.7	.021*	0.5	0.3	0.9	.019*
Gender												
Male (reference)												
Female	0.9	0.7	1.1	0.187	1.1	0.9	1.3	0.243	0.6	0.4	0.8	.002*
Ethnicity												
Chinese (reference)												
Indian	0.7	0.5	1.2	0.185	0.8	0.6	1	0.099	0.5	0.3	1.1	0.082
Malay	2.1	1.5	2.9	<.001**	1.6	1.2	2.1	<.001**	2	1.3	3.2	.003*
Others	1.1	0.6	1.9	0.816	0.8	0.6	1.3	0.401	0.4	0.1	1.4	0.149
Type of Housing												
HDB 1-, 2- or 3-room flat (reference)												
HDB 4- or 5-room flat or executive flat	0.9	0.6	1.3	0.533	1.3	1	1.7	.035*	0.8	0.5	1.4	0.463
Condominiums or other apartments	1	0.7	1.6	0.856	1.4	1	1.9	.038*	0.9	0.5	1.8	0.832
Landed property	0.9	0.5	1.7	0.809	1.2	0.8	1.8	0.469	0.7	0.2	1.8	0.403
Parent’s Highest Education Level												
Below secondary (reference)												
Secondary	0.9	0.5	1.6	0.699	1	0.6	1.6	0.965	0.9	0.4	2.4	0.877
Post-secondary	1.1	0.6	2	0.858	1.1	0.7	1.8	0.753	0.8	0.3	2.2	0.635
Diploma & professional attainment	0.9	0.5	1.6	0.726	0.9	0.5	1.3	0.493	0.9	0.4	2.3	0.873
University	0.8	0.4	1.4	0.347	1	0.6	1.5	0.824	1.1	0.4	2.8	0.798
	ADHD Problems				Oppositional Defiant & Conduct Problems				Comorbidities			
	OR	95%		p value	OR	95%		p value	OR	95%		p value
		CI				CI				CI		
Age Group												
11-12 (reference)												
13-14	1.8	1.1	2.9	.015*	0.9	0.6	1.3	0.534	1.2	1	1.5	0.081
15-16	1.8	1.1	3	.022*	1.3	0.9	1.9	0.169	2	1.5	2.4	<.001
17-18	1.5	0.9	2.5	0.159	1	0.7	1.5	0.991	1.3	1.1	1.7	.015*
Gender												
Male (reference)												
Female	1	0.7	1.4	0.887	0.9	0.7	1.2	0.481	1	0.9	1.2	0.833
Ethnicity												
Chinese (reference)												
Indian	0.8	0.4	1.5	0.41	0.8	0.5	1.3	0.357	0.8	0.6	1	0.058
Malay	1.3	0.8	2.2	0.263	1.7	1.2	2.6	.005*	1.8	1.4	2.3	<.001**
Others	1.3	0.6	2.7	0.542	0.6	0.3	1.4	0.248	0.8	0.5	1.1	0.201
Type of Housing												
HDB 1-, 2- or 3-room flat (reference)												
HDB 4- or 5-room flat or executive flat	1.1	0.7	1.9	0.631	0.8	0.5	1.2	0.288	1.1	0.9	1.5	0.315
Condominiums or other apartments	1.1	0.5	2.1	0.874	0.8	0.4	1.3	0.298	1.3	0.9	1.7	0.155
Landed property	1.1	0.4	2.7	0.915	0.4	0.2	1	0.062	1	0.6	1.5	0.965
Parent’s Highest Education Level												
Below secondary (reference)												
Secondary	1.8	0.6	5.1	0.299	0.9	0.4	1.9	0.737	1	0.6	1.6	0.982
Post-secondary	1.5	0.5	4.7	0.512	0.9	0.4	2	0.77	1.1	0.6	1.8	0.766
Diploma & professional attainment	1.4	0.5	4.1	0.519	1.1	0.5	2.2	0.852	0.9	0.6	1.5	0.77
University	1.2	0.4	3.6	0.708	0.9	0.4	1.9	0.829	1	0.7	1.6	0.914

“*” refers to p values <.001.

**Table 5:** Adjusted sociodemographic correlates for adolescents' problems in the clinical range (CBCL).

	Affective Problems				Anxiety Problems				Somatic Problems			
	OR	95%		p	OR	95%		p	OR	95%		p
		CI		value		CI		value		CI		value
Age Group												
11-12 (reference)												
13-14	1.1	0.8	1.6	0.481	1	0.7	1.2	0.608	0.9	0.6	1.3	0.498
15-16	1.6	1.1	2.2	.013*	0.8	0.6	1.1	0.192	1.2	0.8	1.8	0.42
17-18	1	0.7	1.5	0.88	0.5	0.3	0.6	<.001**	0.9	0.5	1.4	0.512
Gender												
Male (reference)												
Female	1.1	0.8	1.4	0.581	0.7	0.6	0.9	<.001**	1.1	0.8	1.4	0.753
Ethnicity												
Chinese (reference)												
Indian	0.9	0.6	1.4	0.791	0.8	0.5	1.1	0.108	0.9	0.5	1.5	0.633
Malay	1	0.7	1.5	0.964	0.8	0.6	1.1	0.213	1	0.6	1.6	0.948
Others	0.7	0.3	1.4	0.326	1.1	0.7	1.7	0.755	1.4	0.7	2.8	0.315
Type of Housing												
HDB 1-, 2- or 3-room flat (reference)												
HDB 4- or 5-room flat or executive flat	0.7	0.5	1	.033*	1	0.8	1.4	0.804	0.6	0.4	0.9	.015*
Condominiums or other apartments	0.6	0.4	1	.031*	1	0.7	1.4	0.8	0.7	0.4	1.2	0.18
Landed property	0.6	0.3	1.2	0.137	1.1	0.7	1.9	0.711	0.4	0.1	1.1	0.07
Parent’s Highest Education Level												
Below secondary (reference)												
Secondary	0.8	0.4	1.5	0.503	0.9	0.5	1.6	0.76	1.4	0.6	3.1	0.405
Post-secondary	0.9	0.4	1.8	0.682	1.1	0.6	2	0.769	1	0.4	2.5	0.989
Diploma & professional attainment	0.8	0.4	1.6	0.569	0.8	0.5	1.3	0.334	0.8	0.4	1.9	0.678
University	0.7	0.4	1.3	0.214	0.7	0.4	1.1	0.113	0.6	0.3	1.4	0.276
	ADHD Problems				Oppositional Defiant & Conduct Problems				Comorbidities			
	OR	95%		p	OR	95%		p	OR	95%		p
		CI		value		CI		value		CI		value
Age Group												
11-12 (reference)												
13-14	1.1	0.8	1.6	0.51	1	0.7	1.4	0.862	1.1	0.8	1.4	0.695
15-16	0.7	0.5	1.1	0.123	0.7	0.5	1.2	0.199	1	0.7	1.4	0.898
17-18	0.4	0.3	0.7	<.001**	0.5	0.3	0.8	.005*	0.6	0.4	0.8	.002*
Gender												
Male (reference)												
Female	0.4	0.3	0.6	<.001**	0.6	0.4	0.9	.004*	0.7	0.6	0.9	.010*
Ethnicity												
Chinese (reference)												
Indian	0.8	0.5	1.3	0.296	0.4	0.2	0.7	.005*	0.6	0.4	1	.045*
Malay	0.9	0.6	1.4	0.554	0.6	0.4	1	0.072	0.8	0.6	1.2	0.258
Others	0.6	0.3	1.4	0.22	0.6	0.2	1.5	0.271	1	0.6	1.8	0.887
Type of Housing												
HDB 1-, 2- or 3-room flat (reference)												
HDB 4- or 5-room flat or executive flat	0.7	0.5	1	0.079	0.6	0.4	1	.033*	0.6	0.5	0.9	.007*
Condominiums or other apartments	1	0.6	1.6	0.852	0.4	0.2	0.7	.002*	0.6	0.4	0.9	.023*
Landed property	1	0.5	2	0.939	0.4	0.2	1	0.061	0.6	0.3	1.1	0.094
Parent’s Highest Education Level												
Below secondary (reference)												
Secondary	0.9	0.4	2	0.844	0.7	0.3	1.5	0.32	1.2	0.7	2.4	0.443
Post-secondary	1	0.4	2.4	0.994	0.6	0.3	1.6	0.333	1.3	0.6	2.7	0.461
Diploma & professional attainment	1	0.4	2	0.902	0.8	0.3	1.6	0.469	1.1	0.6	1.6	0.746
University	0.6	0.3	1.2	0.15	0.5	0.2	1.2	0.129	0.9	0.4	1.6	0.595

“*” refers to p values <.001.

Based on the YSR, 15-16-year-olds were more likely to have affective (OR, 2.2; 95%CI, 1.6-3.0), anxiety (OR, 1.9; 95%CI, 1.5-2.4), and ADHD problems (OR, 1.8; 95%CI, 1.1-3.0) scores in the clinical range compared to 11-12-year-olds. They were also twice as likely to have comorbidities (95%CI, 1.5-2.4). 13-14-year-olds were more likely to have affective (OR, 1.6; 95%CI, 1.1-2.1) and ADHD problems scores in the clinical range (OR, 1.8; 95%CI, 1.1-2.9) compared to 11-12-year-olds. 17-18-year-olds were more likely to have affective (OR, 1.6; 95%CI, 1.1-2.3) and anxiety problems scores in the clinical range (OR, 1.3; 95%CI, 1.0–1.7) and comorbidities (OR, 1.3; 95%CI, 1.1-1.7), but less likely to have somatic problems (OR, 0.5; 95%CI, 0.3-0.9) compared to 11-12-year-olds. 15-16-year-olds had higher odds than other age groups for affective and anxiety problems, and of having comorbidities.

Females were less likely to have somatic problems than males (OR, 0.6; 95%CI, 0.4-0.8). The odds ratios for Malay adolescents were 2.1 (95%CI, 1.5-2.9) for affective problems, 1.6 (95%CI, 1.2-2.1) for anxiety problems, 2.0 (95%CI, 1.3-3.2) for somatic problems, 1.7 for oppositional defiant/conduct problems (95%CI, 1.2-2.6), and 1.8 (95%CI, 1.4-2.3) for comorbidities. Adolescents staying in a 4-5 room or executive flat (OR, 1.3; 95%CI, 1.0-1.7) and in condominiums or other apartments (OR, 1.4; 95%CI, 1.0-1.9) had higher odds of having anxiety problems in the clinical range compared to those staying in a 1-, 2- or 3-room flat.

Based on the CBCL, 17-18-year-olds had lower odds of having anxiety (OR, 0.5; 95%CI, 0.3-0.6), ADHD (OR, 0.4; 95%CI, 0.3–0.7) and oppositional defiant/conduct problems in the clinical range (OR, 0.5; 95%CI, 0.3-0.8) and comorbidities (OR, 0.6; 95%CI, 0.4-0.8) compared to 11-12-year-olds. 15-16-year-olds had higher odds of having affective problems (OR, 1.6; 95%CI, 1.1-2.2) than 11-12-year-olds.

Females had lower odds of having anxiety (OR, 0.7; 95%CI, 0.6-0.9), ADHD (OR, 0.4; 95%CI, 0.3-0.6) and oppositional defiant/conduct problems in the clinical range (OR, 0.6; 95%CI, 0.4-0.9) and comorbidities (OR, 0.7; 95%CI, 0.6-0.9) than males. Indian adolescents had lower odds of having oppositional defiant/conduct problems in the clinical range (OR, 0.4; 95%CI, 0.2-0.7) and comorbidities (OR, 0.6; 95%CI, 0.4-1.0) than Chinese adolescents. Adolescents staying in a 4-5 room or executive HDB flat had lower odds of having affective (OR, 0.7; 95%CI, 0.5-1.0), somatic (OR, 0.6; 95%CI, 0.4-0.9) or oppositional defiant/conduct problems in the clinical range (OR, 0.6; 95%CI, 0.4-1.0) and comorbidities (OR, 0.6; 95%CI, 0.5-0.9) than those staying in a 1-, 2- or 3-room flat. Adolescents staying in condominiums or other apartments also had lower odds of having affective (OR, 0.6; 95%CI, 0.4-1.0) and oppositional defiant/conduct problems (OR, 0.4; 95%CI, 0.2-0.7) in the clinical range and comorbidities (OR, 0.6; 95%CI, 0.4-0.9) than those staying in a 1-, 2- or 3-room flat.

### 3.4 Association between adolescents’ mental health symptoms and resilience

**Table 6** presents the results of the Spearman rank-order correlation analyses examining the association between adolescent’s clinical mental health symptoms (as measured by the YSR) and resilience levels. Moderate correlations were observed between total resilience scores and internalizing problem scores [rs(3334) = -.50, p <.01], and between total resilience scores and total problem scores [rs(3334) = -.49, p <.01], while the correlation between total resilience scores and externalizing problem scores was relatively weaker [rs(3334) = -.35, p <.01]. The resilience domains that displayed the highest negative correlations with total and internalizing problem scores were: Positive Self-Image/Optimism [rs(3334) = -.54, p <.01; rs(3334) = -.58, p <.01], Personal Control [rs(3334) = -.48, p <.01; rs(3334) = -.52, p <.01], Relationship/Social Support [rs(3334) = -.47, p <.01; rs(3334) = -.47, p <.01] and Emotional Regulation [rs(3334) = -.41, p <.01; rs(3334) = -.43, p <.01].

**Table 6:** Spearman rank-order correlations between adolescents’ problem scores (YSR) and resilience.

	YSR Total	Int	Ext	SYRESS Total	Per/Com	Pos/Op	Rel /Sup	Hum/Pos	Emo/Reg	Spir/Fai	Per Con/Res	PerCtrl	Flex	PosCop
**YSR Total**	**-**														
**Int**	.887^**^	**-**												
**Ext**	.812^**^	.552^**^	**-**											
**SYRESS Total**	-.489^**^	-.501^**^	-.354^**^	**-**											
**Per/Com**	-.323^**^	-.304^**^	-.253^**^	.845^**^	**-**										
**Pos/Op**	-.539^**^	-.581^**^	-.366^**^	.851^**^	.642^**^	**-**									
**Rel /Sup**	-.473^**^	-.467^**^	-.370^**^	.741^**^	.533^**^	.671^**^	**-**								
**Hum/Pos**	-.037^*^	-.125^**^	.043^*^	.519^**^	.420^**^	.377^**^	.311^**^	**-**							
**Emo/Reg**	-.407^**^	-.432^**^	-.284^**^	.748^**^	.550^**^	.572^**^	.457^**^	.345^**^	**-**						
**Spir/Fai**	-.326^**^	-.320^**^	-.264^**^	.782^**^	.642^**^	.638^**^	.547^**^	.317^**^	.505^**^	**-**					
**PerCon/Res**	-.339^**^	-.327^**^	-.252^**^	.869^**^	.752^**^	.669^**^	.615^**^	.465^**^	.632^**^	.629^**^	**-**				
**PerCtrl**	-.481^**^	-.515^**^	-.320^**^	.505^**^	.329^**^	.464^**^	.323^**^	.113^**^	.472^**^	.280^**^	.341^**^	**-**		
**Flex**	-.362^**^	-.386^**^	-.251^**^	.721^**^	.623^**^	.563^**^	.448^**^	.346^**^	.563^**^	.483^**^	.603^**^	.420^**^	**-**	
**PosCop**	-.292^**^	-.248^**^	-.253^**^	.730^**^	.686^**^	.530^**^	.458^**^	.398^**^	.522^**^	.522^**^	.687^**^	.262^**^	.543^**^	**-**

**. Correlation is significant at the 0.01 level (2-tailed).

*. Correlation is significant at the 0.05 level (2-tailed).

Int, Internalizing Problems; Ext, Externalizing Problems; Per/Com, Perseverance/commitment; Pos/Op, Positive Self-Image/Optimism; Rel/Sup, Relationship/Social Support; Hum/Pos, Humour/Positive Thinking; Emo/Reg, Emotional Regulation; Spir/Fai, Spirituality/Faith; PerCon/Res, Personal Confidence/Responsibility; PerCtrl, Personal Control; Flex, Flexibility; and PosCop, Positive Coping.

## 4 Discussion

The YEAR Study is a school-based epidemiology study conducted in a multi-ethnic, Asian city-state that surveys mental health symptoms in school-based adolescents aged 11-18, and thus, its findings on the prevalence of mental health symptoms, the role of resilience and sociodemographic correlates bear important implications for policymaking, preventive efforts and targeted interventions.

### 4.1 Prevalence of mental health symptoms

Based on the YSR, 37.3%, 13.1% and 27.7% of the YEAR Study population scored in the clinical range for internalizing, externalizing and total problems respectively. In comparison to recent studies in Asia, a longitudinal cohort study conducted among Korean students from two middle schools’ representative of the Korean middle school youth population reported prevalence figures of 8.2% and 7.4% for internalizing and externalizing symptoms respectively on the YSR (28). Cui et al. (29)’s national study among Chinese adolescents reported that 17.6% of the study population, representative of the mainland Chinese population, had total problems in the clinical range on the CBCL. No other recent Asian studies were available for comparison. Among other national studies published within the last 10 years, such as in Kosovo (30), Kenya (31), Brazil (32), and Italy (33), the YEAR Study similarly reports the highest prevalence rate of total problem scores and internalizing problem scores in the clinical range. In contrast, the YEAR Study has the lowest prevalence rates of externalizing problems. For the problem subscales, the YEAR study similarly reports the highest prevalence rate in comparison to the studies done in Kosovo, Brazil, and Italy, apart from rule-breaking and aggressive problems, on which the YEAR study holds one of the lowest rates. It should be noted that inter-country comparisons should be interpreted in light of varying contextual factors (e.g., rural vs. urban, different social cultural contexts), and the fact that YEAR study collected data during the COVID-19 pandemic period.

Given Singapore’s unique multi-ethnic and cultural context, some possible explanations of the higher prevalence of internalizing symptoms among Singaporean adolescents may include the high value of academic excellence (from one’s parents or self) inherent in Singaporean, and more broadly, East Asian society. The positive association between academic stress and conditions such as anxiety and depression has been widely explored in past literature (34–36); especially with regards to East Asian societies such as Singapore, China, Korea, Taiwan, Hong Kong and Japan, where the Confucian values of hard work and filial piety hold greater influence (37). In such societies, academic stress often stems from the perception of academic excellence as an obligation towards one’s family, with failure of attainment resulting in familial shame (38). Moreover, when examining sociodemographic correlates, adolescents aged 15-16 had higher odds of having affective and anxiety problems than all other age groups. In Singapore, school-attending adolescents are required to sit for national examinations at age 16, which are perceived as highly crucial in determining one’s post-secondary academic track (Junior College, Polytechnic, Institute of Technical Education, etc.) and subsequent longer term career prospects. Thus, the above finding lends further weight to the role of academic stress in adolescents in predicting internalizing mental health problems.

Another contributing factor may be rooted in the historical narrative and account of Singaporean history legitimised by the Singaporean government, referred to as the ‘Singapore Story’ (39). The historical narrative underscores the vulnerability surrounding Singapore’s early days, characterised by stress and a pervasive drive for survival. Propagated via national education initiatives, this “ideology of survival” (40) – of perseverance and determination to thrive – remains embedded in Singaporean psyche, potentially influencing the Singaporean strive, including academic, and the association with high internalizing problems observed in the adolescent population.

The relatively low prevalence of externalizing problems in Singaporean adolescents compared to adolescents from other countries corroborates estimates presented in earlier studies (41, 42). This finding may be explained by the collectivistic nature of Asian societies which tends to prize behaviour that favours social harmony and interdependence, and frowns upon disruptive behaviour (43). Furthermore, the formal and informal forms of social control (e.g., stringent laws, family values, religious influence, economic stability etc.) inherent in Singaporean society likely contributes to the lower prevalence of externalizing behaviours (44).

### 4.2 Sociodemographic risk

13-14, 15-16 and 17-18-year-olds were all observed to have higher risk of affective and anxiety problems as compared to 11-12-year-olds. 15-16-year-olds had the highest risk compared to the other age groups of affective and anxiety problems, and comorbidities. As explicated above, this finding may reflect the academic stress accompanying a “high-stakes” year. However, as this trend has also been observed in other countries (30, 32), these findings may also be partially explained by the effects of pubertal development. Studies have generally observed depression levels and psychosocial stresses to increase with pubertal development (45). It was also observed that 17-18-year-olds were less likely to have somatic problems scores in the clinical range than 11-12-year-olds, in contrary to findings from previous studies, where somatic symptoms were generally found to increase with age among adolescents (46–48). A study by Feraco and Meneghetti (49) found emotional resilience skills (e.g., stress and impulse regulation) to increase with age (12–19), perhaps indicating the increased capability of older adolescents to understand and express their emotions rather than somatise. However, the Dutch TRAILS Study also reported a decline in somatic symptoms with age (50). Further research is needed to better understand these mixed findings.

In contrast to previous findings (51, 52), females were less likely than males to have somatic problems. Apart from somatic problems, males and females did not significantly differ on the likelihood of having clinical mental health symptoms. A similar finding was observed in the Singapore Mental Health Study (SMHS) – another local epidemiological study on the adult (18 and above) population, where sex was not found to be associated with mental health disorders such as Major Depressive Disorder (MDD) and Generalised Anxiety Disorder (GAD). Historically, females have demonstrated higher risk of MDD (53), but recent findings seem to demonstrate a rising trend of internalizing symptoms among males – as reported by the SMHS, the national prevalence of MDD in males was reported to have increased from 4.3% in 2010 to 5.6% in 2016, perhaps suggesting an increased awareness of mental health struggles among males (9).

Malay adolescents were found to have higher odds in reporting affective, anxiety, somatic and opposition defiant/conduct problems in the clinical range as well as comorbidities, the most pronounced difference being that of affective problems. This finding was observed even after accounting for all other predictors, namely age, sex and our proxy indicators of socioeconomic status (type of housing, parent’s highest qualification). The SMHS also observed higher odds of obsessive-compulsive disorder among Malays (9). However, Malays were also found to have the lowest lifetime risk of at least one mental health disorder (54) and suicide risk (55) compared to other ethnic groups. At this juncture, we do not have any explanation for these mixed findings, and further research is needed to identify the exact cause to further elucidate the unique needs for adolescents of this ethnic group.

Adolescents who lived in a 4-, 5-room or executive flat as well as those who lived in condominiums or other apartments were more likely to have anxiety problems in the clinical range on the YSR. In 2022, the average monthly household income ranged from $9,200-19,936 for those that lived in the above household types (56), representing the middle to higher income categories in Singapore. Although higher socioeconomic status has historically been associated with positive mental health outcomes (57), past literature suggests that parents of a higher socioeconomic status tend to have higher educational expectations of their children (58, 59), which as explicated previously, likely translates to higher academic stress and higher anxiety. Furthermore, relatively affluent children who experience overbearing demands to achieve and parental neglect are thought to be at higher risk of developing anxiety, depression or substance abuse issues (60).

### 4.3 Adolescent vs. parent report

The prevalence rates of mental health symptoms reported by adolescents and their parents using the YSR and CBCL respectively showed differences across the different problems. For instance, 11.2% of parents reported mental health distress of clinical levels in their adolescents, compared to 27.7% based on adolescent report. Similarly, for other scales, prevalence rates derived from parent report were consistently observed to be lower than adolescent-reported rates, this being especially pronounced for depressive and anxiety symptoms. Further analysis such as correlation analyses need to be conducted to interrogate the extent of the discrepancy in reporting.

Differences in reporting between parent and child have been observed in other studies (61–63); studies have identified the quality of the parent-child relationship – parental engagement, communication and acceptance – as one of the stronger predictors of parent-child discrepancies (64, 65). In addition, low mental health literacy among parents may be a contributing factor – a meta-analysis of local studies by Tonsing et al. (66) found the younger generation to possess a greater understanding of mental health conditions, as compared to older generations. Thus, parents could benefit from mental health literacy programs that increase their sensitivity in identifying mental health issues in their children. Moreover, the discrepancies may be the product of certain sociocultural experiences, which colour parental reports. For instance, parents of 17-18-year-olds may be less attuned to the emotional states of their older, more independent adolescents (as compared to 11-12-year-olds).

### 4.4 Resilience and mental health symptoms

Consistent with the extant literature (18, 19, 67), higher resilience scores were observed in this study to be negatively correlated with higher psychopathology score – in a meta-analysis of 60 studies that examined the relationship between resilience and mental health indicators, the mean correlation between resilience and negative mental health indicators was r = -.36 (68). In addition to verifying existing findings on the inextricable link between resilience and mental health, this study identified four pertinent resilience facets: Positive Self-Image/Optimism, Personal Control, Relationship/Social Support and Emotional Regulation, which inversely correlate with YSR scores and could serve as protective factors against mental health symptoms measured by YSR. Further exploration into the protective, moderating effects of these resilience factors and how they can inform intervention targets will be discussed in a subsequent manuscript. For instance, resilience-based parent and teacher interventions were offered to parents and teachers in this current study. Using the theoretical framework underlying the Singapore Youth Resilience Scale (SYRESS), parenting and classroom management tips were shared in the form of a parenting guide and workshops for teachers based on the 10 resilience facets, to inculcate and bolster resilience in adolescents.

## 5 Strengths and limitations

The YEAR study population is reflective of school-going age students in a multi-ethnic Asian population. With its stratified sampling design based on the national student database, its findings on mental health symptoms serve as a reflective benchmark of student mental health in a city-state. Internalizing symptoms are observed to be a key feature of mental health distress in this study population.

This study was conducted in the context of the COVID-19 pandemic, during which social distancing measures was implemented nationwide, and classes were suspended for three months. The pandemic disrupted daily routines and could have amplified the prevalence of anxiety and mood symptoms. The data collection was also suspended when the social distancing measures were enforced, and only resumed after the schools resumed classroom lessons.

The 20.9% response rate of a stratified representative sample may contribute towards a selection bias, as consenting participants may have been more willing to share about their mental health, while those with mental health difficulties may also avoid participating due to perceived stigmatisation from mental health difficulties. This was mitigated by confidentiality and anonymity of responses being explained to participants during the consent-taking process. The consent rate is also in line with other national studies conducted in schools during the COVID-19 pandemic.

Importantly, the respondent population is similar in demographic and educational profile to the national youth population of equivalent age range, though the study population tends to reflect a slightly higher parent’s highest educational level and housing type, which may be protective factors for mental health disorders as found in other studies (57).

Lastly, this study’s cross-sectional design provides a good time-in-point prevalence, but is unable to illustrate cause-effect relationships between study factors.

## 6 Conclusion

The present study illustrates the state of mental health and resilience of Singaporean school-going adolescents aged 11-18, against the backdrop of the COVID-19 pandemic. The relatively high prevalence of internalizing symptoms in Singaporean adolescents, coupled with the reported lower prevalence by parents, warrants greater efforts in increasing literacy among both adolescents and their parents. Furthermore, there exists a need for targeted interventions aimed at those with higher risk of clinical mental health symptoms, such as those belonging to the age category of 15-16. It would be helpful for future prevalence studies to be conducted, so as to determine whether the current prevalence estimates are unique as a result of the pandemic context, or whether they are representative baseline estimates. At the same time, its examination of the associations of mental health symptoms with resilience factors highlights the protective potential of resilience building for children and adolescents.

## Data Availability

The raw data supporting the conclusions of this article will be made available by the authors, without undue reservation.

## Appendix

**Table:** Questionnaires and interview tools administered in the YEAR study.

PHASE 1		
	Adolescent	Parent
Demographics	✓	✓
Psychopathology (YSR, CBCL or BPM-T; Achenbach & Rescorla, 2001)	✓	✓
Singapore Youth Resilience Scale (SYRESS; Broekman, 2011)	✓	
Perceived Stress Scale (PSS; Cohen, Kamarck & Mermelstein, 1994)	✓	
Academic Expectations Stress Inventory (AESI; Ang & Huan, 2006)	✓	
Adolescents’ Medical Conditions		✓
Media Activity Form (MAF; Achenbach, 2018)	✓	✓
Assessment of Identity Development in Adolescents (AIDA; Goth et al., 2012)	✓	
Borderline Personality Feature Scale for Children -11 (BPFSC-11; Sharp et al., 2014)	✓	
Utrecht-Management of Identity Commitments Scale (U-MICS; Crocetti et al., 2010)	✓	
PHASE 2		
	Adolescent	Parent
K-SADS-PL (Kaufman et al., 2016)	✓	✓
Columbia Suicide Severity Rating Scale (CSSRS; Posner et al., 2011)	✓	
Parental Acceptance Rejection Questionnaire (PARQ; Rohner & Khaleque, 2005)	✓	✓
Service Utilisation for Child		✓
Youth Quality of Life Instrument - Short Form (YQOL-SF; Edwards et al., 2002)	✓	
AIDA *(For participants who did not complete in Phase 1)*	✓	
MAF *(For participants who did not complete in Phase 1)*	✓	✓
Patient Health Questionnaire (PHQ-A; Johnson et al., 2002)	✓	
Puberty	✓	
GRID-Hamilton Depression Rating Scale (GRID-HAMD; Williams et al., 2008)	✓	
Saliva (Biomarkers)	✓	
